# Heterogeneous Oriented Structure model of thermoelectric transport in conducting polymers

**DOI:** 10.1038/s41598-023-48353-5

**Published:** 2023-11-30

**Authors:** Patrice Limelette, Nicolas Leclerc, Martin Brinkmann

**Affiliations:** 1 GREMAN UMR 7347, Université de Tours, CNRS, INSA CVL, Parc de Grandmont, 37200 Tours, France; 2 Université de Strasbourg, CNRS, ICPEES UMR 7515, 67087 Strasbourg, France; 3 Université de Strasbourg, CNRS, ICS UPR 22, 67000 Strasbourg, France

**Keywords:** Electronic properties and materials, Thermoelectrics, Polymers

## Abstract

Understanding transport phenomena in conducting polymers (CP) is a main issue in order to optimize their performance and despite intense investigations, the influence of their microstructure remains controversial. By analyzing the thermoelectric measurements performed on highly oriented and non-oriented CP films, we show that an Heterogeneous Oriented Structure (HOSt) model considering both ordered and disordered domains is able to account for the thermoelectric transport in CP. This model unveils the key role of the crystallinity, the anisotropy and the alignment degree of these domains. It points out the importance of the thermal conductivity in the interpretation of the thermopower $\alpha$ and explains the frequently observed electrical conductivity $\sigma$ cut-off in the $\alpha -\sigma$ curves due to the disordered domains. By varying the alignment degree depending on the orientation and the anisotropy according to the face-on or the edge-on polymers conformation, the HOSt model successfully describes the overall measured thermoelectric properties by demonstrating its applicability to a wide variety of both oriented and non-oriented CP.

Conducting polymers are ubiquitous in material science and in plastic electronics^1,2^. They are widely used in the design of organic electronic devices in the form of interfacial layers for charge injection/collection management. These active layers allow a direct conversion of photon and heat into electricity in the case of organic photovoltaic and thermoelectric devices, respectively, or can be used for bioelectronic purposes^3–5^. Recently, doped CP have attracted much attention in the field of thermoelectricity due to the high versatility in material design they offer^4,6,7^, and their low thermal conductivity $\kappa$^8^. The latter is actually a key feature in order to optimize the thermoelectric efficiency as characterized by the dimensionless figure of merit $ZT=\frac{\alpha ^2 \sigma T}{\kappa }$, with the thermopower $\alpha$, the electrical conductivity $\sigma$ and the temperature T. This explains why promising ZT values have been reached in CP such as PEDOT-Tos^9^ and PEDOT-PSS^10^ by getting close to 1. By involving 3 transport coefficients, the figure of merit demonstrates the crucial necessity for a better understanding of transport phenomena in CP in order to further improve their efficiency. Nevertheless, both charge and heat transports remain poorly understood and despite intense research efforts there is so far no consensus on a general transport model. As early identified in the seminal work of Kaiser^11^, the main difficulty originates from the interplay between the complex structure of the CP and their properties. In contrast to either fully crystalline^12^ or amorphous^13^ materials, CP are actually considered to be semicrystalline i.e. they display both kinds of microstructure with crystallographically coherent ordered regions (crystallites or aggregates) coexisting with disordered ones^14^. This has led to raise the question of the influence of the structural disorder on the electronic properties^15^ in such inhomogeneous semicrystalline materials. As a consequence, it is not surprising that the various proposed transport models range from hopping-like by assuming localized charge carriers^16^, to metallic-like by considering nearly or fully delocalized ones^17,18^. Actually, the former models account for the insulating-like temperature dependence of the electrical conductivity for instance, whereas the latter ones are able to describe the metallic-like T-dependence of the thermopower^19,20^ and the observed power law scaling $\alpha \propto \sigma ^{-1/4}$ as a function of doping^17,18,21–24^. This also explains why a recent phenomenological model has been proposed in order to capture both localized and delocalized transport simultaneously^25^. At variance with these models, Kaiser^20,21^ proposed a qualitative alternative approach involving the electrical resistances of both the crystallites and amorphous regions, based on the early recognition of their heterogeneous structure^14,19,26^. Kaiser succeeded in relating the macroscopic electrical conductivity to phenomenological parameters accounting for the unknown geometrical factors characterizing ordered and disordered regions^11,20^. Herein, we extend this approach by proposing an Heterogeneous Oriented Structure (HOSt) model that accounts not only for the semicrystallinity but also for the anisotropy of the CP films. The HOSt model considers three key parameters: the crystallinity ratio, the alignment degree and the intrinsic transport anisotropy within crystallites in order to describe the macroscopic electrical and thermal conductivity and the thermopower in both oriented and non-oriented CP. This model is tested by using a wide collection of thermoelectric data measured in thin films of poly(2,5-bis(3-alkyl-2-thienyl)thieno[3,2-b]thiophene), PBTTT-C$_{12}$ or -$^8$O (Fig. 1a and “Methods section”), with strong acceptor molecules e.g. 2,3,5,6-tetrafluoro-tetracyanoquinodimethane (F$_4$TCNQ), 1,3,4,5,7,8-hexafluoro-tetracyano-naphtho-quinodimethane (F$_6$TCNNQ) or ferric chloride FeCl$_3$ (Fig. 1b)^22,27,28^. High temperature rubbing (Fig. 1c) is an elegant method to impart high orientation and order to conducting polymers that can be readily doped. Such oriented and non-oriented CPs are then ideal to probe anisotropic transport properties and to asses the validity of transport models by performing cross-correlations between various transport coefficients (charge conductivity and Seebeck coefficient) along and perpendicular to the polymer chain direction. As a results, it is shown how this model also accounts for the thermoelectric properties measured in non-oriented doped-PBTTT by demonstrating its wide applicability.

**Figure 1:**
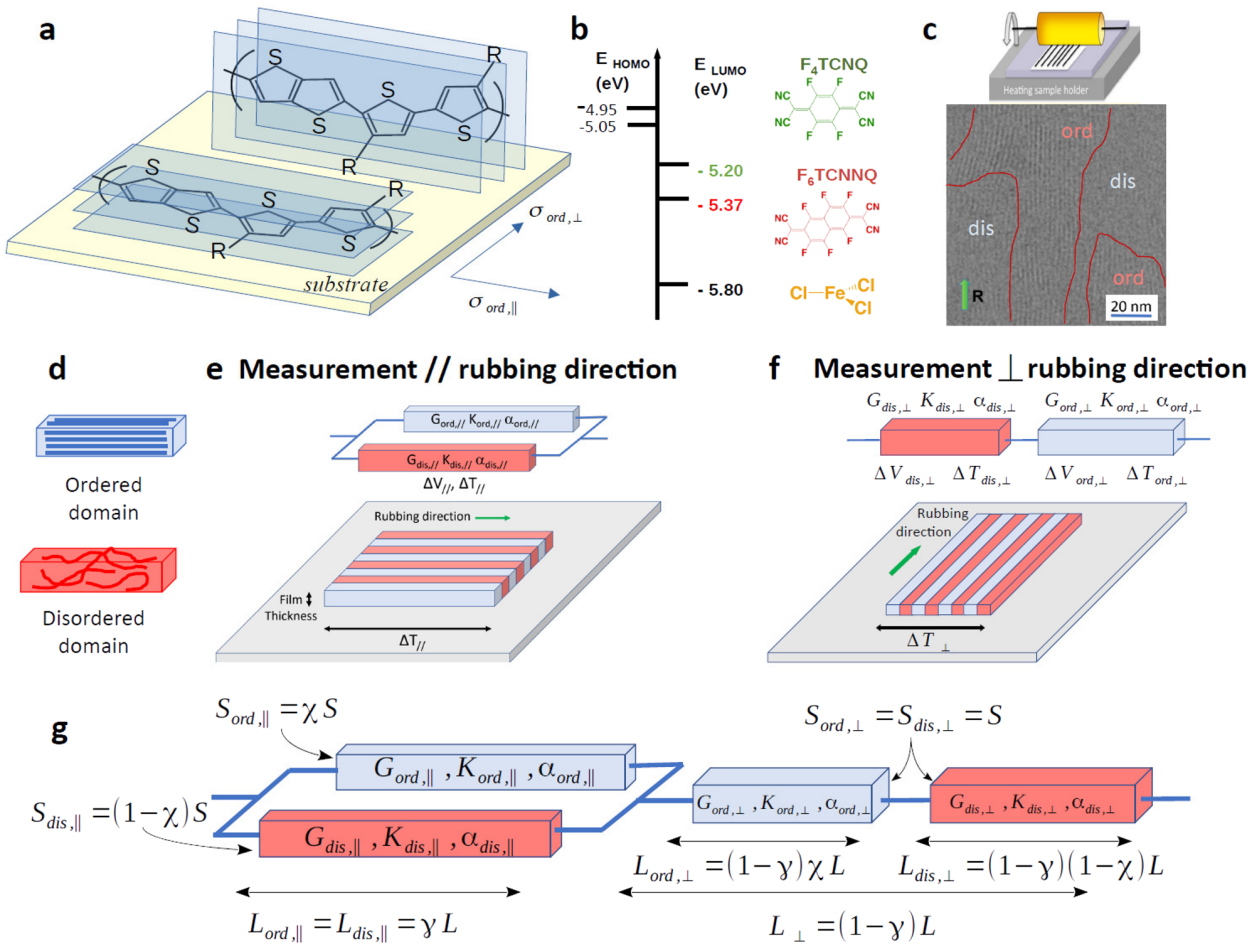
(**a**) Chemical structure of the PBTTT-R polymers (R = C$_{12}$H$_{25}$) in the ordered regions where the blue area indicates the direction of the conjugated chain backbone with the corresponding electrical conductivity. An electrical anisotropy $\beta$ is then expected in the direction perpendicular to the latter with $\sigma _{ord,\perp } = \frac{\sigma _{ord,\parallel }}{\beta }$. Two contact planes are here displayed, with the face-on orientation where the $\pi$-stacking is normal to the substrate and the edge-on orientation where the $\pi$-stacking is parallel to the substrate. These two orientations are here expected to coexist in the investigated oriented films whereas in non-oriented ones the edge-on packing is usually favored by leading to a lower transport anisotropy. (**b**) p-type dopants as well as the energetic diagram comparing the HOMO (Highest Occupied Molecular Orbital) position of the polymers and the LUMO (Lowest Unoccupied Molecular Orbital) of the dopants. (**c**) Orientation method of the polymers by high temperature rubbing and HR-TEM image of an oriented PBTTT-C$_{12}$ film showing the semicrystalline structure with the coexistence of ordered and disordered domains consistent with the proposed model^23,29–31^. (**d**) Schematic illustration of the semicrystalline oriented conducting polymers made of alternating disordered (red) and ordered (blue) domains that run in (**e**) parallel to the rubbing direction and (**f**) alternate perpendicularly to the rubbing direction. The equivalent electrical circuits are represented when the current/temperature gradient is parallel (||) or perpendicular ($\perp$) to the rubbing direction. (**g**) The generalization to non-oriented conducting polymers is realized by connecting in series both parallel (**e**) and perpendicular (**f**) configurations with the corresponding geometrical factors which are related to the crystallinity $\chi$ and alignment $\gamma$ ratios as defined in the text. $\gamma$ = 1 corresponds to a perfect parallel configuration (**e**) and $\gamma$ = 0 to a perpendicular one (**f**).

## Results

### HOSt model

The proposed model consists basically in introducing 3 parameters in order to describe the complex structure of the conducting polymers in terms of crystallinity, anisotropy and preferred orientation of ordered and disordered domains. The crystallinity accounts for the proportion of ordered or crystalline domains in the CP. It is then defined as the ratio between the volume of the ordered regions over the total one as $\chi =\frac{V_{ord}}{V_{tot}}$ and can vary from 0 in the case of completely amorphous polymer up to 1 if the CP is perfectly crystallized. The anisotropy results from the quasi one-dimensionnal molecular structure of the polymer backbone (Fig. 1a) which should influence the charge transport depending on the stacking of the chains in the ordered domains. Accordingly, a transport anisotropy $\beta$ is thus introduced as $\sigma _{ord,\perp }= \frac{\sigma _{ord,\parallel }}{\beta }$ with $\beta >1$ if the chains are oriented in the direction parallel to the rubbing and then $\sigma _{ord,\parallel } > \sigma _{ord,\perp }$. The third parameter describes the preferred orientation of the domains and is closely related to the experimental method of high temperature rubbing (Fig. 1c) which allows to align the ordered domains (Fig. 1d) in the direction of the rubbing. This leads to introduce an alignment factor $\gamma$ which accounts for the degree of orientation along the rubbing direction such as if $\gamma =1$ the ordered domains are parallel to the latter direction. This corresponds to the situation ideally represented in Fig. 1e for which both heat and charge fluxes operate in the parallel direction. If they are perpendicular to the domains as in Fig. 1f the alignment is then ideally considered to be $\gamma =0$. Moreover, the alignment parameter can be used to build an effective non-oriented medium by considering that in such a case the mosaicity of the domains orientation can be projected onto both oriented configurations, namely parallel and perpendicular. The alignment parameter becomes therefore a potentially continuous variable between 0 and 1 consistent with the previously discussed limiting cases as allowed thanks to the geometrical factors shown in Fig. 1g. As depicted, the effective length of the parallel component as well as the perpendicular one can be varied according to $\gamma$ by preserving the overall constant length while the crystallinity changes the proportion of ordered and disordered domains by keeping constant the overall section. The crystallinity, the anisotropy and the alignment allow then to build a versatile model based on realistic key parameters.

### Transport coefficients in the HOSt model

In order to describe the thermoelectric transport properties corresponding to an heterogeneous medium as depicted in Fig. 1e and f, the global transport coefficients need to be related to those associated to the disordered and ordered domains. As shown in the “Methods section”, the electrical G or thermal K conductances are summed in the parallel configuration and the resulting thermopower $\alpha _{\parallel }$ is a sum of each contribution, $\alpha _{dis,\parallel }$ and $\alpha _{ord,\parallel }$, from the disordered and ordered domains respectively weighted by the relative electrical conductance. In the perpendicular configuration, the inverse of the conductances are summed and each thermopower contribution, $\alpha _{dis,\perp }$ and $\alpha _{ord,\perp }$, is weighted by the relative thermal conductance.

As it will be justified by the experimental results in the forthcoming section, the thermopower can be further simplified if the contribution originating from the disordered regions can be neglected such as $\alpha _{dis,\perp } \approx \alpha _{dis,\parallel } \approx 0$. To go one step further, the electrical (thermal) conductance G (K) must be related to the electrical (thermal) conductivity of the ordered and disordered domains $\sigma _{ord}$ and $\sigma _{dis}$ ($\kappa _{ord}$ and $\kappa _{dis}$) which characterize the heterogeneous medium. This implies to take into account the geometrical factors as shown in Fig. 1g since by definition G = $\sigma$S/L and K = $\kappa$S/L where L is the length and S the section of the sample. At this point, we must distinguish the dimensions of ordered and disordered domains which are characterized by typical mesoscopic dimensions as seen in the HR-TEM image of Fig. 1c. Also the crystallinity ratio $\chi$ can be introduced according to Fig. 1g by assuming that it modifies the sections between ordered and disordered regions in parallel direction as $S_{ord,\parallel }= \chi S$ and $S_{dis,\parallel } = (1-\chi ) S$ with a constant length as $L_{ord,\parallel } = L_{dis,\parallel } = L$ ($\gamma = 1$). In the perpendicular direction, $\chi$ changes the length as $L_{ord,\perp }=\chi L$ and $L_{dis,\perp }=(1-\chi ) L$ since the section is kept constant as $S_{ord,\perp }= S_{dis,\perp } = S$ ($\gamma = 0$). By doing so, the total volume remains conserved as well as the overall both length and section. Accordingly, in the direction parallel to the chains/rubbing:

1 $$\begin{aligned} \sigma _{\parallel } = \chi \sigma _{ord,\parallel } + (1-\chi ) \sigma _{dis,\parallel } \;\;\;\;\; \;\;\;\;\; \alpha _{\parallel } \approx \frac{ \chi \sigma _{ord,\parallel } }{\left( \chi \sigma _{ord,\parallel } + (1-\chi ) \sigma _{dis,\parallel } \right) } \alpha _{ord,\parallel } \end{aligned}$$

In the direction perpendicular to the rubbing, we obtain:

2 $$\begin{aligned} \sigma _{\perp } = \frac{ \sigma _{ord,\perp } \sigma _{dis,\perp }}{(1-\chi ) \sigma _{ord,\perp }+ \chi \sigma _{dis,\perp } } \;\;\;\;\; \;\;\;\;\; \alpha _{\perp } \approx \frac{ \chi \kappa _{dis,\perp } }{\left( (1-\chi ) \kappa _{ord,\perp } + \chi \kappa _{dis,\perp } \right) } \alpha _{ord,\perp } \end{aligned}$$

These are the two basic equations describing the correlations between $\alpha$ and $\sigma$ for an oriented heterogeneous material as a function of the properties of ordered and disordered domains, with the anisotropy $\beta$ previously defined as $\sigma _{ord,\perp }= \frac{\sigma _{ord,\parallel }}{\beta }$. The definitions of the thermal conductivities $\kappa _{\parallel ,\perp }$ follow those of the electrical conductivities. According to Eqs. (1) and (2), the non-oriented case shown in Fig. 1g can then be straightforwardly characterized with its $\gamma$-dependent transport coefficients as below (see the “Methods section”).

3 $$\begin{aligned} \alpha = \gamma \frac{ \kappa }{ \kappa _{~ \parallel } } \alpha _{ \parallel } + (1-\gamma ) \frac{ \kappa }{ \kappa _{ \perp } } \alpha _{ \perp } \;\;\;\;\; \;\;\;\;\; \sigma = \frac{ \sigma _{\parallel } \sigma _{ \perp } }{ (1-\gamma ) \sigma _{\parallel } + \gamma \sigma _{ \perp } } \;\;\;\;\; \;\;\;\;\; \kappa = \frac{ \kappa _{\parallel } \kappa _{ \perp } }{ (1-\gamma ) \kappa _{\parallel } + \gamma \kappa _{ \perp } } \end{aligned}$$

Thus, this set of 3 equations describes the thermoelectric properties of non-oriented conducting polymers, as well as those of oriented ones in both parallel ($\gamma =1$) and perpendicular ($\gamma =0$) directions by including variable crystallinity ratio $\chi$, anisotropy degree $\beta$ and alignment $\gamma$. We will now confront this model with experimental data gained for highly oriented PBTTT films.

**Figure 2:**
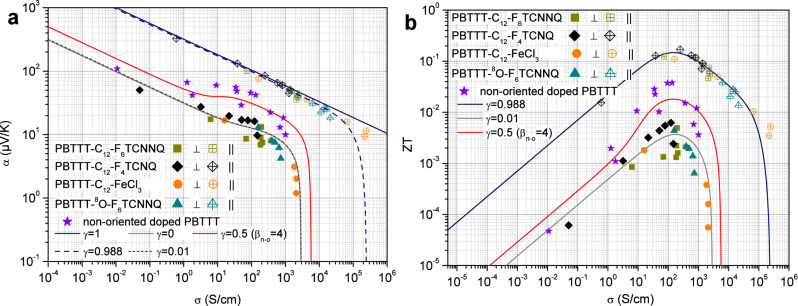
(**a**) Room temperature thermopower $\alpha$ as a function of the electrical conductivity $\sigma$ in a double logarithmic scale in non-oriented and oriented PBTTT, in both parallel (open symbols) and perpendicular (full symbols) directions. The solid lines with $\gamma$ = 1 and $\gamma$ = 0 result from the HOSt model in perfectly oriented cases according to Eqs. (1) and (2). The dashed lines ($\gamma$ = 0.988 and $\gamma$ = 0.01) illustrate the effect of nearly perfectly oriented cases according to equations (3). The used transport parameters are summarized in Table 1 according to Fig. 3a, b and the analysis explained in the text with the crystallinity $\chi$=0.55 and the anisotropy $\beta _o$ = 10. Note that the small deviation between the HOSt model and the data measured in the parallel direction in the high conductivity regime can be removed by fine tuning the alignment degree. The HOSt model also accounts for the thermoelectric properties measured in non-oriented doped-PBTTT according to equations (3) with the same crystallinity $\chi$ = 0.55, an alignment $\gamma$ = 0.5 in agreement with the orientations mosaicity and a lower anisotropy $\beta _{n-o}$ = 4 than in oriented configurations due to the edge-on packing. **b**, The thermoelectric figure of merit ZT is plotted as a function of the electrical conductivity in the different investigated configurations, non-oriented and oriented, by using the thermal conductivity as inferred from the HOSt model and summarized in Table 1. Note that the observed behaviors typically display an enhancement of the optimum ZT by a factor 5 between the perpendicular configuration and the non-oriented one and by another factor 5 between the latter and the parallel configuration reaching the maximum value ZT$\approx$0.15.

Transport properties have been measured in non-oriented and oriented PBTTT films at room temperature over a wide range of doping levels for various dopants (see Fig. 1b) and for PBTTTs with different side chains (C$_{12}$ and C$_7$-O-C$_4$). As pointed out in a recent study, the observed scaling laws are largely independent of the chemical nature of the dopants and of the side chains whereas they depend strongly on the ordering of backbones and their in-plane alignment obtained by high-T rubbing^32^. Such a large set of experimental data offers a unique opportunity to probe the validity of this model. The overall measurements and the modeling are summarized in Fig. 2 where the thermopower is in particular plotted as a function of the electrical conductivity in a double logarithmic scale in Fig. 2a.

As seen in Fig. 2a, the $\alpha -\sigma$ scalings are different in oriented CP films in the directions parallel and perpendicular to the polymer chains. In oriented PBTTT films $\alpha _{\parallel } \propto \sigma _{\parallel }^{-1/4}$ in the chain direction whereas in the direction perpendicular to the chains^23,33^, the thermopower $\alpha _{\perp }$ first follows a similar power law as $\sigma _{\perp }^{-1/4}$ for low conductivities but departs from this behavior by tending to 0 at a cut-off conductivity value of the order of 2.5 $\times$10$^3$ S/cm. The $\alpha -\sigma$ correlation curve in non-oriented films lies somewhere in-between these two cases by displaying an intermediate more complex behavior which will be discussed thereafter.

## Discussion

Let us first analyze the results in the direction parallel to the polymer chains. As frequently observed in a wide variety of conducting polymers^11,22,23^, the thermopower measured in the parallel direction varies as a function of the electrical conductivity according to a power law which is a typical signature of a degenerate or metallic state^17,18,34^. The unconventional exponent 1/4 has been quite recently explained by the scattering of Dirac-like quasiparticles by unscreened ionized impurities^18,34,35^. Since this power law appears well-defined over the whole range of conductivity (0.5–10$^5$ S/cm), it means that the Eq. (1) should apply in a purely parallel configuration as a good approximation with $\sigma _{ord,\parallel }>> \sigma _{dis,\parallel }$ and then $\alpha _{\parallel } \approx \alpha _{ord,\parallel }$ and $\sigma _{\parallel } \approx \chi \sigma _{ord,\parallel }$. This implies that the thermoelectric properties measured in such a configuration are essentially those of the oriented ordered regions. Thus, the following relation 4 successfully describes the thermoelectric properties of oriented PBTTT conducting polymer in the parallel direction (Fig. 2a, $\gamma =1$).

4 $$\begin{aligned} \alpha _{ord,\parallel } = \frac{\pi ^2}{ 3 } \frac{k_B}{e} 4 \left( \frac{\sigma _{ord,\parallel }}{\sigma _{0,\parallel }} \right) ^{-1/4} \qquad \Rightarrow \qquad \alpha _{\parallel } \approx \frac{\pi ^2}{ 3 } \frac{k_B}{e} 4 \left( \frac{\sigma _{\parallel }}{\chi \sigma _{0,\parallel }} \right) ^{-1/4} \end{aligned}$$

This provides a preliminary analysis leading to $\sigma _{0,\parallel } \approx 0.013$ S/cm if one considers that disordered and ordered phases are nearly equally present in the sample, with $\chi \approx 0.55$. $\sigma _{0,\parallel }$ appears higher than in other CP^18^ in accordance with the expected larger mobility in PBTTT^36,37^. It is worth mentioning that the high conductivity values reached in the parallel direction likely results from the very careful sample preparation as well as the transport measurements performed in a glovebox as detailed in the “Method section”. Also, the values as 10$^4$ S/cm which have already been measured in other CP such as PPV^20^ (PolyPhenyleneVinylene) should correspond to a mobility of the order of 16 cm$^2$/Vs if one considers a charge density of the order of 4 $\times$ 10$^{21}$ cm$^{-3}$. The latter mobility is indeed higher than the usually reported field effect mobility ranging between 0.1 and 1 cm$^2$/Vs in PBTTT^38^ but it is not unrealistic if it is compared with the ones measured in organic semiconductors which can reach for instance 43 cm$^2$/Vs^39^.

Let us now consider the results in the direction perpendicular to the chains as described by Eq. (2) according to Fig. 1f. In such a configuration, the thermopower is expected to be the sum of each contribution weighted by their relative thermal conductance (see the “Methods section”). In the high conductivity regime, the thermal conductance of the ordered regions is expected to be very high due to the contribution of charge carriers that adds to the lattice contribution according to the Wiedemann-Franz law^34,35,40–42^. Moreover, the electrical conductivity of ordered domains is much larger than that of disordered domains and $K_{ord,\perp }>> K_{dis,\perp }$. This implies that the thermopower $\alpha _{\perp }$ measured in this regime should tend towards the contribution from disordered regions and hence $\alpha _{\perp } \approx \alpha _{dis,\perp } + \frac{K_{dis,\perp }}{K_{ord,\perp }}\alpha _{ord,\perp } \rightarrow \alpha _{dis,\perp }$ (see the “Methods section”). On the other hand, the thermopower measured for the highest conductivity values (> 10$^3$ S/cm) is strongly decreasing down to nearly 1 $\mu V/K$ in Fig. 2a without showing any saturation. This suggests that the thermopower of the disordered regions is lower than the latter value and can be neglected i.e. $\alpha _{dis,\perp } \approx 0$. This justifies the approximation made in Eqs. (1) and (2). From a physical point of view, a negligible thermopower may be related to the disordered nature of these regions favoring a broad energetic landscape for charge carriers consistent with a nearly constant electronic density of states. According to the Mott formula of the thermopower in variable range hopping regime, such a density of states should then imply a vanishing thermopower contribution in agreement with our conclusions^13^.

**Figure 3:**
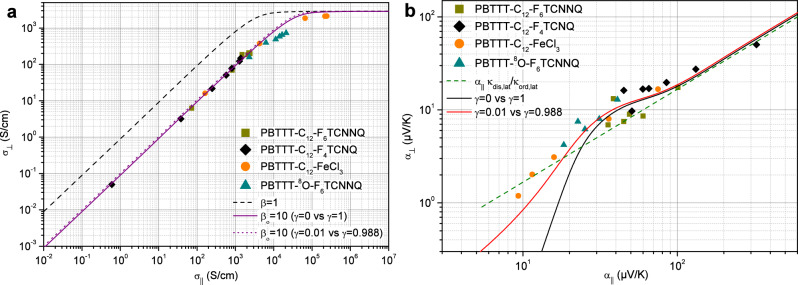
(**a**) Variation of the electrical conductivity measured in the perpendicular direction $\sigma _{\perp }$ as a function of $\sigma _{\parallel }$ measured in the parallel one. The solid line is the expected behavior according to Eqs. (1) and (2) ($\gamma$ = 1 and $\gamma$ = 0) in the HOSt model with $\chi$ = 0.55, and the transport parameters summarized in Table 1. The dashed line with $\beta$ = 1 shows as a comparison the influence of the anisotropy of the ordered regions, the other parameters being the same. The dotted line with $\beta _o$ = 10 demonstrates that there is no expected real difference in the case of nearly perfect orientations ($\gamma$ = 0.988 and $\gamma$ = 0.01) according to Eq. (3). The slope, the cross-over and the saturation value combined with the scaling in Fig. 2a allow to infer electrical transport parameters. **b**, Variation of the thermopower measured in the perpendicular configuration $\alpha _{\perp }$ as a function of $\alpha _{\parallel }$ measured in the parallel one. The dashed line is the expected linear behavior when only considering the effect of the lattice thermal conductivity of both ordered and disordered regions as $\alpha _{\perp } \approx \frac{ \kappa _{dis,lat} }{ \kappa _{ord,lat} } \alpha _{\parallel }$ with $\kappa _{ord,lat} \approx 6 \kappa _{dis,lat}$. The observed departure from the linearity is fully explained by considering the electronic contribution to the thermal conductivity according to the Wiedmann-Franz law $\kappa _{el}=\frac{\pi ^2}{3} \left( \frac{k_B}{e}\right) ^2 \sigma T$ as shown with the solid lines. The black one considers the perfectly oriented cases ($\gamma$ = 1 and $\gamma$ = 0) according to Eqs. (1) and (2), and the red one, the nearly perfectly oriented cases ($\gamma$ =0.988 and $\gamma$ = 0.01) according to Eq. (3). In contrast to Fig. 3a, the latter is here found to improve the adequacy with the experimental results. The observed main slope and the departure from the linearity allow to characterize with the scaling in Fig. 2a the thermal transport parameters.

In order to further analyze the thermoelectric transport in the perpendicular direction, it appears meaningful to correlate first the electrical conductivity measured in the latter direction with the one measured in the parallel direction for the same doping as performed in Fig. 3a. Actually, since $\sigma _{ord, \parallel }>> \sigma _{dis, \parallel }$ and $\sigma _{ord, \perp }>> \sigma _{dis, \perp }$ it follows according to equations 1 and 2 that the electrical conductivities are essentially given by $\sigma _{\parallel } \approx \chi \sigma _{ord, \parallel }$ and $\sigma _{\perp } \approx \chi \sigma _{dis, \perp }/(1-\chi )$. This implies that the variation displayed in Fig. 3a mainly represents the dependence of $\sigma _{dis,\perp }$ with $\sigma _{ord,\parallel }$, with a clear proportionality at low doping followed by a saturation at high doping. The observed linearity suggests that the doping influences both the disordered and ordered regions but the former being characterized by lower charge carriers mobility, the resulting electrical conductivity is lower than in the ordered regions. In contrasts to the latter, the electrical conductivity in the disordered regions turns out to be limited at high doping likely due to to their amorphous structure which prevents the charge carriers from delocalizing and seems to saturate up to a value close to the Mott minimum metallic conductivity^13^. Such a behavior appears then consistent with a variation as $\sigma _{dis,\perp } = \sigma _{dis,\perp }^{max} \left( 1-e^{-\sigma _{ord,\perp }/\sigma _{c-o,\perp }} \right)$ with $\sigma _{ord,\perp }=\sigma _{ord,\parallel }/\beta =\sigma _{\parallel }/\chi \beta$. The saturation of $\sigma _{\perp }$ in Fig. 3a allows to determine $\sigma _{dis,\perp }^{max}$. The analysis of both the slope and the cross-over leading to the saturation provide, with the scaling in Fig. 2a, the anisotropy $\beta _o = 10$ and the cristallinity $\chi \approx 0.55$, with the values $\sigma _{c-o,\perp } \approx 4000$ S/cm and $\sigma _{dis,\perp }^{max} \approx 1300$ S/cm. As shown in Fig. 3a, these values agree quite well with the experimental results according to Eqs. (1) and (2) by considering either perfect orientations with $\gamma =1$ and $\gamma =0$ or nearly perfect ones with $\gamma \approx 0.988$ and $\gamma \approx 0.01$ according to Eq. (3).

So, the inferred anisotropy indicates an easy conduction direction along the polymer backbone in contrast to the perpendicular direction which is characterized by a significant reduction. Such an anisotropy appears consistent with the molecular architecture and the values as discussed in the literature^15,38,43,44^. The retrieved crystallinity ratio accounts for the heterogeneous structure of the polymer in agreement with the typical values of the order of 50$\%$ as reported^14,15^. It supports the assumption that rubbed films consist of a network of highly oriented and interconnected domains, as assumed in the HOSt model.

The same kind of analysis can also be performed by using now the thermopower measured in both parallel and perpendicular configurations for the same doping as shown in Fig. 3b. As previously discussed the thermopower of the disordered regions can be neglected in Eq. (2) and $\alpha _{\perp }$ is then expected to be proportional to $\alpha _{ord,\perp }$. Due to the considered anisotropy, the latter should vary as $\left( \sigma _{ord,\perp }/\sigma _{0,\perp } \right) ^{-1/4}$ according to Eq. (4) but since the transport parameter $\sigma _{0,\perp }$ caries all the possible anisotropies^17,18^, one infers that $\alpha _{ord,\perp } = \alpha _{ord,\parallel } \approx \alpha _{\parallel }$. Thus, it follows that $\alpha _{\perp } \approx \frac{ \chi \kappa _{dis,\perp } }{\left( (1-\chi ) \kappa _{ord,\perp } + \chi \kappa _{dis,\perp } \right) } \alpha _{\parallel } \approx \frac{ \chi \kappa _{dis,\perp } }{(1-\chi ) \kappa _{ord,\perp } } \alpha _{\parallel }$ if one considers that the thermal conductivity in the ordered regions is higher than in the disordered ones. On the other hand, the thermal conductivity involves a lattice contribution and an electronic component, doping dependent, which is usually given by the Wiedmann-Franz law $\kappa _{el}=\frac{\pi ^2}{3} \left( \frac{k_B}{e}\right) ^2 \sigma T$^34^. Therefore, in the low doping regime one expects the latter being much lower than the lattice component and then that the thermopower in the perpendicular configuration is basically given by the ratio of lattice thermal conductivity as $\alpha _{\perp } \approx \frac{ \chi \kappa _{dis,lat} }{(1-\chi ) \kappa _{ord,lat} } \alpha _{\parallel }$, where the subscript $\perp$ has been dropped for simplicity. This relation explains the main linear behavior seen in Fig. 3b between $\alpha _{\perp }$ and $\alpha _{\parallel }$ which is highlighted by the dashed line corresponding to the lattice thermal conductivity ratio $\kappa _{dis,lat}/ \kappa _{ord,lat} =1/6$. Note that the higher thermopower values correspond to the lower electrical conductivity values measured in the low doping regime which justify the expected linear behavior between $\alpha _{\perp }$ and $\alpha _{\parallel }$. Interestingly, the observed departure from this linearity can be quite straightforwardly understood by considering the electronic contribution to the thermal conductivity. According to the values of $\alpha _{\parallel }$ three regimes can actually be identified in Fig. 3b. In the low doping regime where $\alpha _{\parallel }>100$ $\mu$V/K, the electronic component of the thermal conductivity can be neglected due to the low electrical conductivity, $\kappa _{dis,lat}>> \kappa _{dis,el,\perp }$ and $\kappa _{ord,lat}>> \kappa _{ord,el,\perp }$, and it follows as previously discussed that $\alpha _{\perp } \approx \frac{ \chi \kappa _{dis,lat} }{(1-\chi ) \kappa _{ord,lat} } \alpha _{\parallel }$ and the variation is purely linear. When the electrical conductivity increases with the doping, the electronic contribution to the thermal conductivity of the disordered regions is no more negligible and there is an enhancement of $\alpha _{\perp }$ with respect to the previous linear behavior as $\alpha _{\perp } \approx \frac{ \chi (\kappa _{dis,lat}+\kappa _{dis,el,\perp }) }{(1-\chi ) \kappa _{ord,lat} } \alpha _{\parallel }$ seen within the range $20<\alpha _{\parallel }<100$ $\upmu$V/K. At this stage, there is no significant electronic contribution from the ordered regions because $\kappa _{ord,lat} = 6 \kappa _{dis,lat}$ and the lattice component remains higher than the electronic one. When the doping further increases and $\alpha _{\parallel }< 20$ $\upmu$V/K, $\kappa _{dis,el,\perp }$ becomes constant due to the saturation of the electrical conductivity $\sigma _{dis,\perp }$ as shown in Fig. 3a while $\sigma _{ord,\perp }$ and then $\kappa _{ord,el,\perp }$ still increase. $\kappa _{dis,\perp }$ being constant and $\kappa _{ord,\perp }$ increasing, it results that the thermopower decreases faster than the linear behavior given by the lattice thermal conductivity ratio as $\alpha _{\perp } \approx \frac{ \chi \kappa _{dis,\perp } }{(1-\chi ) (\kappa _{ord,lat}+\kappa _{ord,el,\perp }) } \alpha _{\parallel }$. This interpretation fully agrees with the experimental results displayed in Fig. 3b as well supported by Eqs. (1) and (2) in the case of perfect configurations with $\gamma = 1$ and $\gamma =0$, by considering the lattice thermal conductivity $\kappa _{ord,lat} \approx 0.2$ W/m/K and $\kappa _{dis,lat} \approx 0.033$ W/m/K. As a strong check, the agreement is even more improved if one considers according to Eq. (3) that the experimental orientations are only nearly prefect with $\gamma = 0.988$ and $\gamma =0.01$ for the parallel and the perpendicular configurations respectively. Even if the specific inferred values of lattice thermal conductivity should be considered with some caution, one must emphasize that they appear quantitatively consistent with the usual low values of thermal conductivity ranging typically between 0.2 and 1 W/m/K, depending on the doping, as reported in literature for conducting polymers^9,10,41,42,45^.

**Table 1:** Summary of the inferred transport parameters as used in Fig. 2 for both non-oriented ($\gamma =0.5$, $\chi =0.55$, $\beta _{n-o}=4$) and oriented PBTTT, in parallel ($\gamma =1-0.988$, $\chi =0.55$, $\beta _o=10$) and perpendicular ($\gamma =0-0.01$, $\chi =0.55$, $\beta _o=10$) directions.

Ordered domains		Disordered domains					
$\sigma _{0,\parallel }$	$\kappa _{ord,lat}$	$\sigma _{dis,\perp }^{max}$	$\sigma _{c-o,\perp }$	$\sigma _{dis,\parallel }^{max}$		$\sigma _{c-o,\parallel }$	$\kappa _{dis,lat}$
(S/cm)	(W/m/K)	(S/cm)	(S/cm)	(S/cm)		(S/cm)	(W/m/K)
				Oriented	Non-oriented		
0.013	0.2	1300	4000	0.8	57	13	0.033

Note that the electrical conductivity in the disordered domains are assumed to vary as $\sigma _{dis,\parallel ,\perp } = \sigma _{dis,\parallel ,\perp }^{max} \left( 1-e^{-\sigma _{ord,\parallel ,\perp }/\sigma _{c-o,\parallel ,\perp }} \right)$ namely linearly at low doping up to the maximum value $\sigma _{dis,\parallel ,\perp }^{max}$ reached above a cross-over value $\sigma _{c-o,\parallel ,\perp }$.

As a result, the use of the several inferred transport parameters allows to successfully reproduce the scaling of the thermopower as a function of the electrical conductivity in both parallel and perpendicular configurations in Fig. 2a, according to Eqs. (1) and (2) with $\gamma = 1$ and $\gamma =0$ or Eq. (3) with $\gamma = 0.988$ and $\gamma =0.01$ in the case of nearly prefect parallel and perpendicular configurations respectively. In agreement with the electrical conductivity in Eq. (1), a small disordered component has been considered such as $\sigma _{dis,\parallel } = \sigma _{dis,\parallel }^{max} \left( 1-e^{-\sigma _{ord,\parallel }/\sigma _{c-o,\parallel }} \right)$ for the sake of consistency even if it remains quite negligible with $\sigma _{dis,\parallel }^{max} \approx 0.8$ S/cm and $\sigma _{c-o,\parallel } \approx 13$ S/cm. These values appears however of interest because they suggest that the rubbing procedure, besides favoring a preferred orientation of the ordered regions, likely stretches the disordered regions in the rubbing direction by making the conduction path less efficient. This appears therefore consistent with the higher electrical conductivity found in the perpendicular direction compared to the one deduced in the parallel direction with $\sigma _{dis,\perp }>>\sigma _{dis,\parallel }$. These conclusions seem to be even more supported if one considers now the case of the non-oriented doped PBTTT as reported in Fig. 2a. Since the polymer is non-oriented, one must assume that the orientations of both ordered and disordered regions are randomly distributed. Due to the wide number of regions, it seems then relevant to consider equal proportions of parallel and perpendicular components according to the Fig. 1g and Eq. (3), namely an alignment degree $\gamma = 0.5$. Therefore, the HOSt model allows to successfully describe the observed complex scaling between the thermopower and the electrical conductivity in Fig. 2a by using the previously determined transport parameters as summarized in Table 1 with the exception of disordered parallel electrical component $\sigma _{dis,\parallel }^{max}$ and the anisotropy. It is actually found that the former is indeed enhanced with $\sigma _{dis,\parallel }^{max}\approx 57$ S/cm in agreement with the fact that the rubbing stretches the disordered regions along its direction as previously suggested. If the conduction mechanism in the disordered regions is related to the presence of polymers chains bridging two ordered regions, the latter enhanced value appears then consistent with the expected smaller disordered regions in non-oriented CP. On the other hand, the anisotropy of the ordered regions appears to be lower than in the oriented cases with $\beta _{n-o} \approx 4$. This could also result from the high temperature rubbing since it favors coexisting edge-on and face-on orientations whereas it is known that the edge-on configuration is predominant in non-oriented PBTTT^44^. Therefore, the found lower anisotropy $\beta _{n-o} \approx 4$ is consistent with the expected higher charge carriers mobility in the $\pi$-stacking direction^15^ due to the edge-on contact planes. Beyond this successful modeling, a careful examination of Eq. (3) shows approximately that $\sigma \propto \sigma _{dis,\perp }$ whereas $\alpha \propto \alpha _{ord,\parallel } \kappa _{dis,\perp }/\kappa _{ord,\perp }$. This means that in non-oriented CP, the measured electrical conductivity is mainly governed by the disordered regions whereas the thermopower is strongly influenced by the ordered ones. By considering that $\sigma _{dis,\perp }^{max}$ carries the insulating-like temperature dependence of the disordered regions and that $\alpha _{ord,\parallel }$ is metallic-like, the HOSt model explains then the coexistence between the insulating-like electrical conductivity and the metallic-like thermopower usually measured in CP.

**Figure 4:**
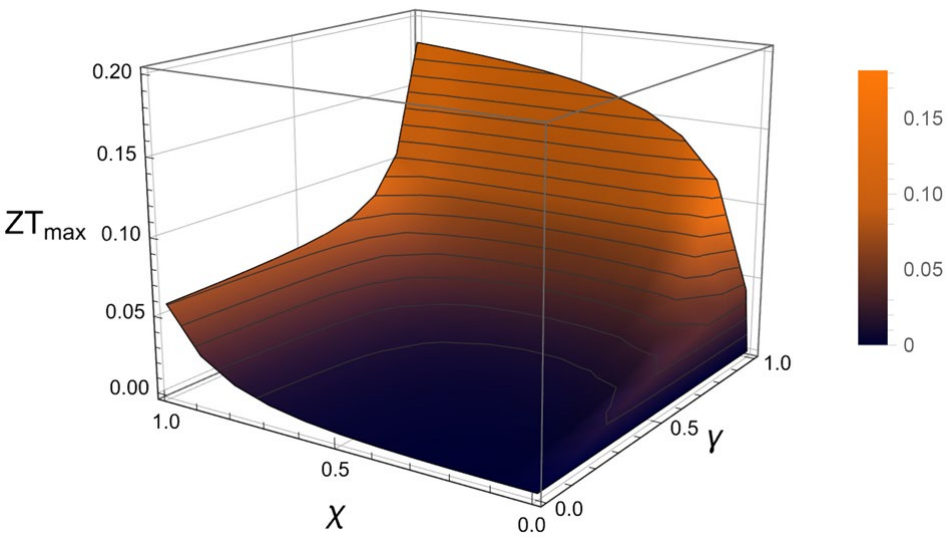
Variation of the maximum thermoelectric figure of merit ZT$_{max}$ as a function of the crystallinity ratio $\chi$ and the alignment degree $\gamma$ as expected in the frame of the HOSt model with the inferred transport parameters ($\beta _o$ = 10). Even if the thermoelectric optimum appears when both crystallinity and alignment tend to 1, figure of merit higher than 0.1 can be reached whatever the crystallinity ($\chi$> 0.05) if $\gamma$> 0.9, and higher than 0.05 whatever the alignment if $\chi$> 0.9. A noticeable enhancement can be seen around $\chi \approx 0.1$ which increases with the alignment by exceeding 0.05 if $\gamma$> 0.8.

Furthermore, the HOSt model allows to infer the thermal conductivity which is necessary to deduce the dimensionless figure of merit $ZT=\frac{\alpha ^2 \sigma T}{\kappa }$ in both non-oriented and oriented configurations as displayed in Fig. 2b. By revealing a maximum ZT in the parallel configuration reaching 0.15 at room temperature, these results show that the thermoelectric performance is enhanced by a factor 5 between the perpendicular configuration and the non-oriented one and by another factor 5 between the latter and the parallel configuration. With a calculated maximum figure of merit of the same order of magnitude than the highest reported to date in conducting polymers^9,10^, this result highlights that the alignment process provides a route toward high thermoelectric efficiency as shown in Fig. 4. On the other hand, the various correlations performed in the frame of the reported analysis are done between the transport coefficients themselves and not as a function of the doping. Therefore, whatever the doping efficiency our approach and the model remain valid as long as the dopants don’t alter significantly the polymers structure. Furthermore, the model is suitable to describe the thermoelectric transport properties measured in conducting polymers prepared by other processes. As a matter of fact, the HOSt model is successfully used to describe the thermoelectric properties measured in the non-oriented PBTTT film which is not prepared by high temperature rubbing. Nevertheless, some inferred characteristics should depend on the preparation processes which influence the alignment degree and the crystallinity ratio first, but also the transport parameters characterizing the disordered regions.

The developed model with its ability to characterize quantitatively the complex thermoelectric transport in conducting polymers, either oriented or non-oriented, demonstrates that a correct description requires to take into account both their heterogeneous structure and anisotropy in order to be able to explain their specific transport characteristics. By involving both disordered and ordered domains, the HOSt model also reconciles the apparent discrepancy^17–24^ between the metallic-like and insulating-like properties usually measured in conducting polymers. Finally, the successful description of the thermoelectric properties measured in other non-oriented CP, as shown in the Supplementary information file, demonstrates the wide applicability of the HOSt model which should even more promote the development and the use of conducting polymers in the field of organic electronics.

## Methods section

### Samples preparation and measurements.

The preparation of the oriented films of PBTTT-C$_{12}$ and PBTTT-$^8$O follows the methodology presented in our previous publications^28,32,44^. The synthesis of the polymers poly(2,5-bis(3-dodecyl-2-yl)thieno[3,2-b]thiophene) (PBTTT-C$_{12}$) and (poly(2,5-bis(7-butoxyheptyl-2-yl)thieno[3,2-b]thiophene) (PBTTT-$^8$O) has also been previoulsy reported^28,44^. Dopants such as FeCl$_3$ and F$_4$TCNQ were obtained from Sigma Aldrich and TCI companies, respectively. The dopant F$_6$TCNNQ was synthesized following a known protocol^32^. Importantly, the choice of the dopant for a given p-type polymer is dictated by the relative positions of the polymer’s HOMO and the dopant’s LUMO (Fig. 1b). The approach by sequential doping of CP is particularly relevant since it allows to tune independently the structure/crystallinity of the CP and the subsequent doping step by introduction of dopant molecules in the polymer host^38,44,46^. Moreover, controlling crystallinity and/or alignment (Fig. 1c) are effective means to enhance TE performances of the doped films^44,47–51^. On the other hand, anhydrous solvents from Aldrich such as ortho-dichlorobenzene (ODCB) and acetonitrile were used without further purification. Sodium Poly(styrene) sulfonate (NaPSS) (1000 kg/mol) was supplied by Sigma Aldrich. In brief, a polymer film is first prepared by doctor blading a 20–30 mg/ml solution in ODCB at 150$^\circ$C onto NaPSS-coated glass substrates. The NaPSS films are sacrificial layers to recover the oriented PBTTT films by floating onto distilled water and transfer to the substrates with electrodes or TEM copper grids. NaPSS films are prepared by spin-coating a 10 mg/ml aqueous solution at 3000 RPM on clean glass substrates (microscope slides). Rubbing is performed with a home-made machine that applies a rotating cylinder (600 RPM) covered with a microfiber cloth at 2–3 bar onto the polymer coated substrates maintained at the rubbing temperature. The temperature of the films is allowed to equilibrate for approximately 1 min prior to rubbing. The alignment is ascertained by Polarized light Microscopy and further quantified by polarized UV-vis-NIR spectroscopy using a Cary 5000 spectrometer. After floating of the PBTTT films onto substrates, they are left to dry in ambient (1 hr) prior to degassing in a primary vacuum upon transfer to the glovebox (Jacomex, P(O$_2$)<1 ppm and P(H$_2$O)<1 ppm) for doping. Dopant solutions are prepared readily before their use and conductivity and Seebeck coefficients are measured also shortly after doping on dry films. Doping using the incremental concentration method is used by dipping the PBTTT films into the dopant solution for approximately 1 min for each concentration^44^. No rinsing step was used. The semicrystalline morphology of CPs such as PBTTT (Fig. 1c) has been thoroughly investigated and AFM as well as High Resolution TEM evidenced highly ordered domains with typical dimensions of the order of a few tens of nanometers in the chain direction (see Fig. 1c) surrounded by disordered (amorphous) domains^23,29,30^. The setup for four point probe conductivity and Seebeck measurements is described in detail elsewhere^51^. All conductivity and Seebeck coefficients were measured at ambient temperature in a glovebox.

### Heterogeneous Oriented Structure model

In the case where the current and the temperature gradient are perpendicular to the rubbing direction, the effective electrical circuit is then composed of two elements in series with one corresponding to the disordered regions and the other to the ordered ones (see Fig. 1e and f). Each of them are characterized by their own transport coefficients, a priori distinct from the ones in parallel direction, with the electrical and the thermal conductances G and K, and the thermopower $\alpha _{dis,\perp }$ and $\alpha _{ord,\perp }$ for disordered and ordered domains, respectively. In this case, both voltages and temperatures differences are summed such as $\Delta V_{\perp } = \Delta V_{dis,\perp } + \Delta V_{ord,\perp } = \alpha _{dis,\perp } \Delta T_{dis,\perp }+ \alpha _{ord,\perp } \Delta T_{ord,\perp } = \alpha _{\perp } \Delta T_{\perp }$ which leads to an overall perpendicular thermopower as a sum of two contributions originating from disordered and ordered regions weighted by the corresponding relative temperatures differences. The heat current being conserved, $|I_{H,\perp }|= K_{\perp } \Delta T_{\perp } = K_{dis,\perp } \Delta T_{dis,\perp } = K_{ord,\perp } \Delta T_{ord,\perp }$ and the relative temperatures differences are given by $\frac{\Delta T_{dis/ord,\perp }}{\Delta T_{\perp }}=\frac{K_{\perp }}{ K_{dis/ord,\perp }}$. The inverse of the conductances being summed, it results that the thermopower can be written as a sum of each contribution weighted by the relative thermal conductance.

5 $$\begin{aligned} K_{\perp } = \frac{K_{ord,\perp } K_{dis,\perp }}{ K_{ord,\perp }+ K_{dis,\perp } } \;\;\;\;\; \;\;\;\;\; \alpha _{\perp } = \frac{ K_{ord,\perp } }{K_{ord,\perp } + K_{dis,\perp }} \alpha _{dis,\perp } + \frac{ K_{dis,\perp } }{K_{ord,\perp } + K_{dis,\perp } } \alpha _{ord,\perp } \end{aligned}$$

In the case where the temperature gradient is oriented parallel to the rubbing (chain) direction, ordered and disordered regions are now in parallel. It follows that all the voltages as well as the temperatures differences are equal and then $\Delta V_{\parallel } = \Delta V_{dis,\parallel }= \Delta V_{ord,\parallel }$ and $\Delta T_{\parallel } = \Delta T_{dis,\parallel }= \Delta T_{ord,\parallel }$. Thus, the charge current is $|I_{C,\parallel }|= G_{\parallel } ( \Delta V_{\parallel } + \alpha _{\parallel } \Delta T_{\parallel }) = G_{dis,\parallel } ( \Delta V_{\parallel } + \alpha _{dis,\parallel } \Delta T_{\parallel }) + G_{ord,\parallel } ( \Delta V_{\parallel } + \alpha _{ord,\parallel } \Delta T_{\parallel })$ . Aside from recovering that the conductances are summed, the thermopower can be expressed as a sum of each contribution from disordered and ordered domains weighted by the relative electrical conductance.

6 $$\begin{aligned} G_{\parallel } = G_{ord,\parallel } + G_{dis,\parallel } \;\;\;\;\; \;\;\;\;\; \alpha _{\parallel } = \frac{ G_{dis,\parallel } }{G_{ord,\parallel } + G_{dis,\parallel } } \alpha _{dis,\parallel }+ \frac{ G_{ord,\parallel } }{G_{ord,\parallel } + G_{dis,\parallel } } \alpha _{ord,\parallel } \end{aligned}$$

Equations (5) and (6) provide the basis of the HOSt model that allow to describe the transport properties of oriented conducting polymers in the direction respectively perpendicular and parallel to the chain direction (rubbing direction). On the other hand, it is assumed that these two limit cases also provide the two basic components which can be found in any non-oriented conducting polymer since the latter consist in a complex arrangement of the two kinds of regions in parallel and in series. Therefore, the simplest way to build an effective medium with both components, parallel and perpendicular, accounting for the lack of macroscopic preferred orientation is to connect the Fig. 1e, f in series electrically as shown in Fig. 1g. The relative weight of each component is then given by the geometrical factors of the transport coefficients themselves. So, due to the series electrical connection the same treatment which has led to Eq. (5) can be performed by introducing the total transport coefficients G, K and $\alpha$. The latter can then be related to the parallel and perpendicular transport coefficients defined in Eqs. (5) and (6), according to the inferred following relations (7).

7 $$\begin{aligned} K = \frac{K_{\perp } K_{\parallel }}{ K_{\perp }+ K_{\parallel } } \;\;\;\;\; \;\;\;\;\; G = \frac{G_{\perp } G_{\parallel }}{ G_{\perp }+ G_{\parallel } } \;\;\;\;\; \;\;\;\;\; \alpha = \frac{ K_{\perp } }{K_{\perp } + K_{\parallel }} \alpha _{\parallel } + \frac{ K_{\parallel } }{K_{\perp } + K_{\parallel } } \alpha _{\perp } \end{aligned}$$

The geometrical factors shown in Fig. 1g allow then to deduce Eq. 3.

### Supplementary Information

Supplementary Information 1.

Supplementary Information 2.

## Data Availability

Source data are provided with this paper. The collected datasets are included in this published article, and additional datasets that have been analyzed are from the cited literature. A Supplementary information file proposes an extended discussion on the transport parameters used in the frame of the HOSt model and shows its successful application to other non-oriented conducting polymers. Furthermore, in order to assist in the dissemination of the HOSt model, we provide a Supplementary LibreOffice worksheet (Supplementary HOSt model Interactive Monitor). For convenience, any additional datasets generated and analyzed during the current study will also be made available from the corresponding authors upon reasonable request.
