# Supplementary material for: Heterogeneous Oriented Structure model of thermoelectric transport in conducting polymers
Source: Sci Rep. 2023 Nov 30;13:21161. doi: 10.1038/s41598-023-48353-5 (PMC10689499; doi:10.1038/s41598-023-48353-5)
Supplement: Supplementary file 1 — Supplementary Information 1. [file 41598_2023_48353_MOESM1_ESM.pdf]

Supplementary Information

Heterogeneous Oriented Structure model of thermoelectric transport in conducting polymers

Patrice Limelette[1], Nicolas Leclerc[2] and Martin Brinkmann[3]

(1) GREMAN UMR 7347, Université de Tours, CNRS, INSA CVL, Parc de Grandmont, F-37200 Tours, France

(2) Université de Strasbourg, CNRS, ICPEES UMR 7515, F-67087 Strasbourg, France

(3) Université de Strasbourg, CNRS, ICS UPR 22, F-67000 Strasbourg, France

An extended discussion is provided in this supplementary file in order to give more insight into the transport parameters of the disordered domains as used in the HOSt model. The forthcoming discussion should be of particular interest in order to be able to model the thermoelectric properties measured in non-oriented conducting polymers (CP) while those of the oriented CP remain unknown. In this context, the four cases detailed below allow to describe the effects of the transport parameters of the disordered domains with the maximum value σ_{dis}^{max} and the cross-over one σ_{c-o} above which σ_{dis}^{max} is reached, in both parallel and perpendicular components. Assumptions are then proposed in order to relate these parameters to the location of the dopants and the shape anisotropy of the disordered domains. Finally, a successful application of the HOSt model to other conducting polymers is shown by using the data provided in literature. It is found in particular that even in the PEDOT-Tos, this model succeeds in describing the thermoelectric properties including the experimental figure of merit.

Transport parameters of the disordered domains

The HOSt model involves several transport parameters in order to account for the electronic properties of both disordered and ordered domains and then it may appear difficult to implement especially in non-oriented CP. In order to facilitate its use, typical cases are thereafter described which allow to identify more easily the range of the required parameters from the experimental results by plotting the thermopower as a function of the electrical conductivity in a conventional way.

Actually, four cases can be distinguished rather straightforwardly in Fig. S1 (γ=0.5, χ=0.5, β=1) depending on the transport parameters of both the parallel and perpendicular components of the disordered domains. If $\sigma_{dis,\parallel}^{max} \ll \sigma_{c-o,\parallel} \ll \sigma_{dis,\perp}^{max} \ll \sigma_{c-o,\perp}$ as in Fig. S1a (yellow curve), the thermopower mainly varies in the low conductivity regime as $\alpha \propto \sigma^{-1/4}$ and then it decreases gradually when the maximum value of the perpendicular component $\sigma_{dis,\perp}^{max}$ is reached. The behavior is the same in the high conductivity regime if $\sigma_{c-o,\parallel} \ll \sigma_{dis,\parallel}^{max} \ll \sigma_{dis,\perp}^{max} \ll \sigma_{c-o,\perp}$ but a crossover takes place at low conductivity due to the influence of the parallel component (red curve). In such a condition, the latter component increases faster than the electrical conductivity of the ordered domains themselves by implying a shortcut and then a decrease of the thermopower. This downshift disappears when the maximum value $\sigma_{dis,\parallel}^{max}$ is reached because σ_{ord} can thus exceeds it. The situation is quite different if one compares the first discussed behavior to the case

represented in Fig. S1b with $\sigma_{dis,\parallel}^{max} \ll \sigma_{c-o,\parallel} \ll \sigma_{c-o,\perp} \ll \sigma_{dis,\perp}^{max}$ (blue curve). Now this is the perpendicular component which increases faster than σ_{ord} and then a substantial excess of thermopower is provided by an enhanced thermal conductivity due to the perpendicular component of the disordered domains. This explains the upshift observed in Fig. S1b as well as the bump at high conductivity. On the other hand, an interesting behavior appears if $\sigma_{c-o,\parallel} \ll \sigma_{dis,\parallel}^{max} \ll \sigma_{c-o,\perp} \ll \sigma_{dis,\perp}^{max}$ as shown in Fig. 1c (dark blue curve). This is likely the most surprising case for which both perpendicular and parallel components of the disordered domains increase faster at low doping than σ_{ord} . It follows that the conventional power law behavior of the thermopower seems nearly lost due to the bump at high conductivity and the shortcut at low conductivity implying a nearly constant thermopower over a sizable range of conductivity. The Fig. S1d can help to understand this behavior by comparing this case to the one displayed in Fig. S1b too. So, by considering the shape of the experimental curve α–σ and the fact the maximum value of the electrical conductivity observed experimentally is limited by $\sigma < \sigma_{dis,\perp}^{max} / [(1-\chi)(1-\gamma)]$ according to equations (3), the range of the transport parameters can then be roughly inferred.

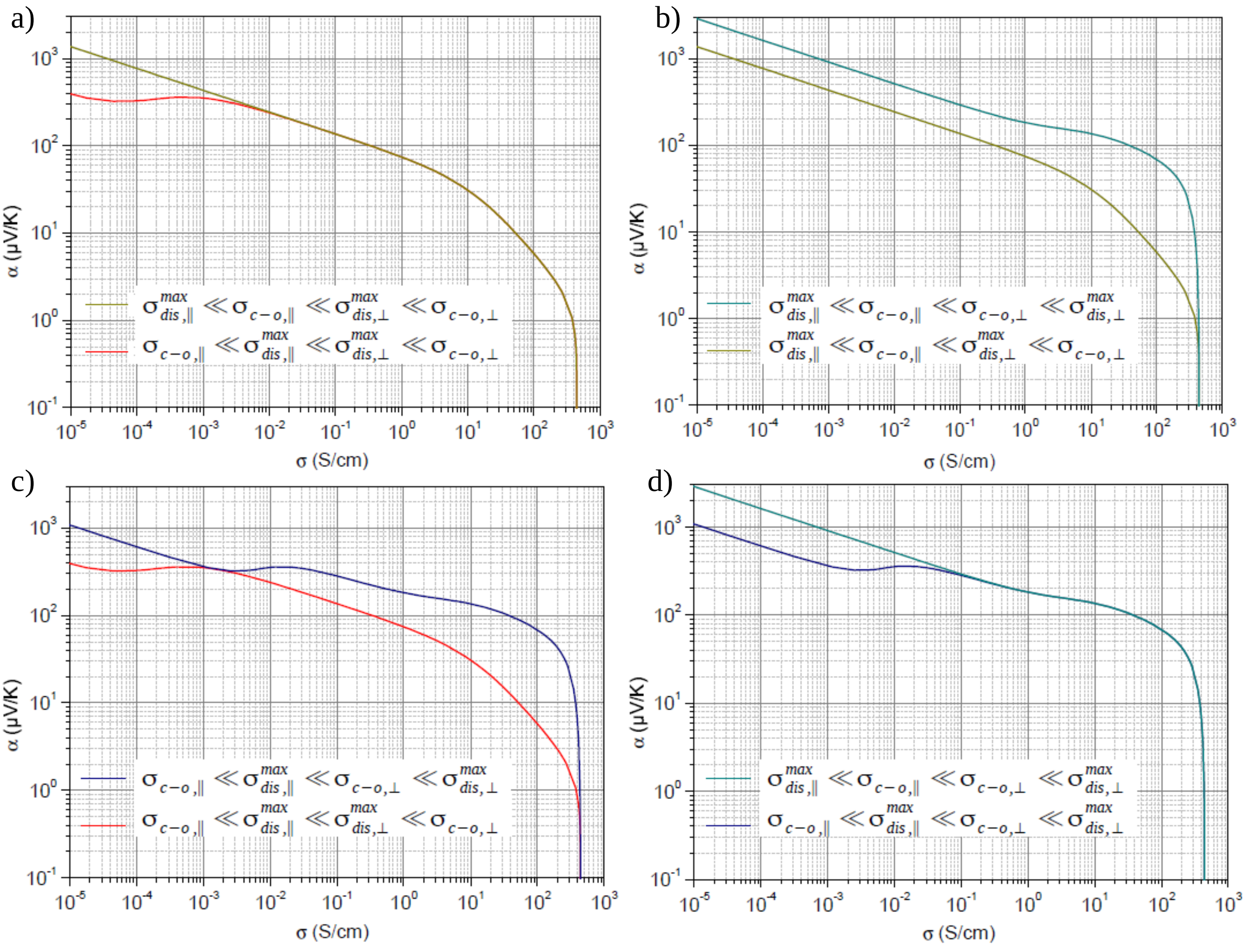

Figure S1: Thermopower as a function of the electrical conductivity according to the HOSt model with γ=0.5, χ=0.5 and β=1 in the selected cases (**a**) $\sigma_{dis,\parallel}^{max} \ll \sigma_{c-o,\parallel} \ll \sigma_{dis,\perp}^{max} \ll \sigma_{c-o,\perp}$ and $\sigma_{c-o,\parallel} \ll \sigma_{dis,\parallel}^{max} \ll \sigma_{dis,\perp}^{max} \ll \sigma_{c-o,\perp}$, (**b**) $\sigma_{dis,\parallel}^{max} \ll \sigma_{c-o,\parallel} \ll \sigma_{c-o,\perp} \ll \sigma_{dis,\perp}^{max}$ and $\sigma_{dis,\parallel}^{max} \ll \sigma_{c-o,\parallel} \ll \sigma_{dis,\perp}^{max} \ll \sigma_{c-o,\perp}$, (**c**) $\sigma_{c-o,\parallel} \ll \sigma_{dis,\parallel}^{max} \ll \sigma_{c-o,\perp} \ll \sigma_{dis,\perp}^{max}$ and $\sigma_{c-o,\parallel} \ll \sigma_{dis,\parallel}^{max} \ll \sigma_{dis,\perp}^{max} \ll \sigma_{c-o,\perp}$, (**d**) $\sigma_{dis,\parallel}^{max} \ll \sigma_{c-o,\parallel} \ll \sigma_{c-o,\perp} \ll \sigma_{dis,\perp}^{max}$ and $\sigma_{c-o,\parallel} \ll \sigma_{dis,\parallel}^{max} \ll \sigma_{c-o,\perp} \ll \sigma_{dis,\perp}^{max}$.

Meaning of the transport parameters of the disordered domains

Locations of the dopants. In the selected cases previously discussed, the situation with $\sigma_{c-o} \ll \sigma_{dis}^{max}$ is in particular considered for the parallel or the perpendicular component. Since we have assumed a doping dependence such as $\sigma_{dis} = \sigma_{dis}^{max}\left(1 - e^{-\sigma_{ord}/\sigma_{c-o}}\right)$, the electrical conductivity is expected to vary linearly at low doping as $\sigma_{dis} \approx \sigma_{ord} \sigma_{dis}^{max} / \sigma_{c-o}$ and then $\sigma_{dis} > \sigma_{ord}$ if $\sigma_{c-o} \ll \sigma_{dis}^{max}$. It means that the electrical conductivity in the disordered domains increases faster than in the ordered domains up to the saturation value when the doping increases. Whereas this behavior could appear at first sight surprising, it could actually indicate that the dopants are preferentially located in the disordered domains (amorphous regions) which should lead to a higher doping efficiency in these regions compared to the ordered ones at low doping. At the contrary if $\sigma_{c-o} \gg \sigma_{dis}^{max}$, the electrical conductivity in the disordered domains increases more slowly than in the ordered domains up to the saturation value when the doping increases. Therefore, this suggests that the dopants are in this case preferentially located in the ordered domains which should lead to a higher doping efficiency in these regions compared to the disordered ones.

Shape anisotropy of the disordered domains. On the other hand, one can assume that the magnitude of $\sigma_{dis,\perp}^{max}$ with respect to $\sigma_{dis,\|}^{max}$ might be a consequence of the shape anisotropy of the disordered domains. If the width of the disordered domains is much smaller than their extension, a higher doping efficiency could occur in the perpendicular component compared to the parallel one leading to $\sigma_{dis,\|}^{max} \ll \sigma_{dis,\perp}^{max}$. Inversely if the width of the disordered domains is much larger than their extension, the higher doping efficiency should occur in the parallel component compared to the perpendicular one and then, $\sigma_{dis,\|}^{max} \gg \sigma_{dis,\perp}^{max}$. In the case of disordered domains without such a shape anisotropy, one should expect $\sigma_{dis,\|}^{max} \approx \sigma_{dis,\perp}^{max}$.

As a result, it appears that the transport parameters of the disordered domains could provide some information concerning their shape anisotropy and the location of the dopants, and reciprocally, the knowledge of these informations could influence the choice of transport parameters in the model.

Application of the HOSt model to other non-oriented conducting polymers

An application of the HOSt model is also provided below by using previously reported data in other CP such as P3HT-$FeCl_3$ [1], PA [2-7], PBTTT [8, 9] and PEDOT-Tos [10]. It is shown in Fig. S2a-d that it can suitably account for the correlation between the measured thermopower and the electrical conductivity in these 4 non-oriented CP with the transport parameters summarized in the Table 1. Since these CP are non-oriented, the values of the transport parameters determined in our investigations (see the main ext) for the non-oriented samples have been used as starting values (Table 1, *PBTTT**) by considering too the shape of the $\alpha-\sigma$ curve according to the previous discussion. Then, the transport characteristics of the disordered domains have been mainly adjusted, without changing the alignment, the crystallinity, the anisotropy and the lattice thermal conductivity. It follows that these parameters should be considered with caution since as discussed in the main text, a more precise characterization requires for instance thermoelectric measurements in oriented compounds.

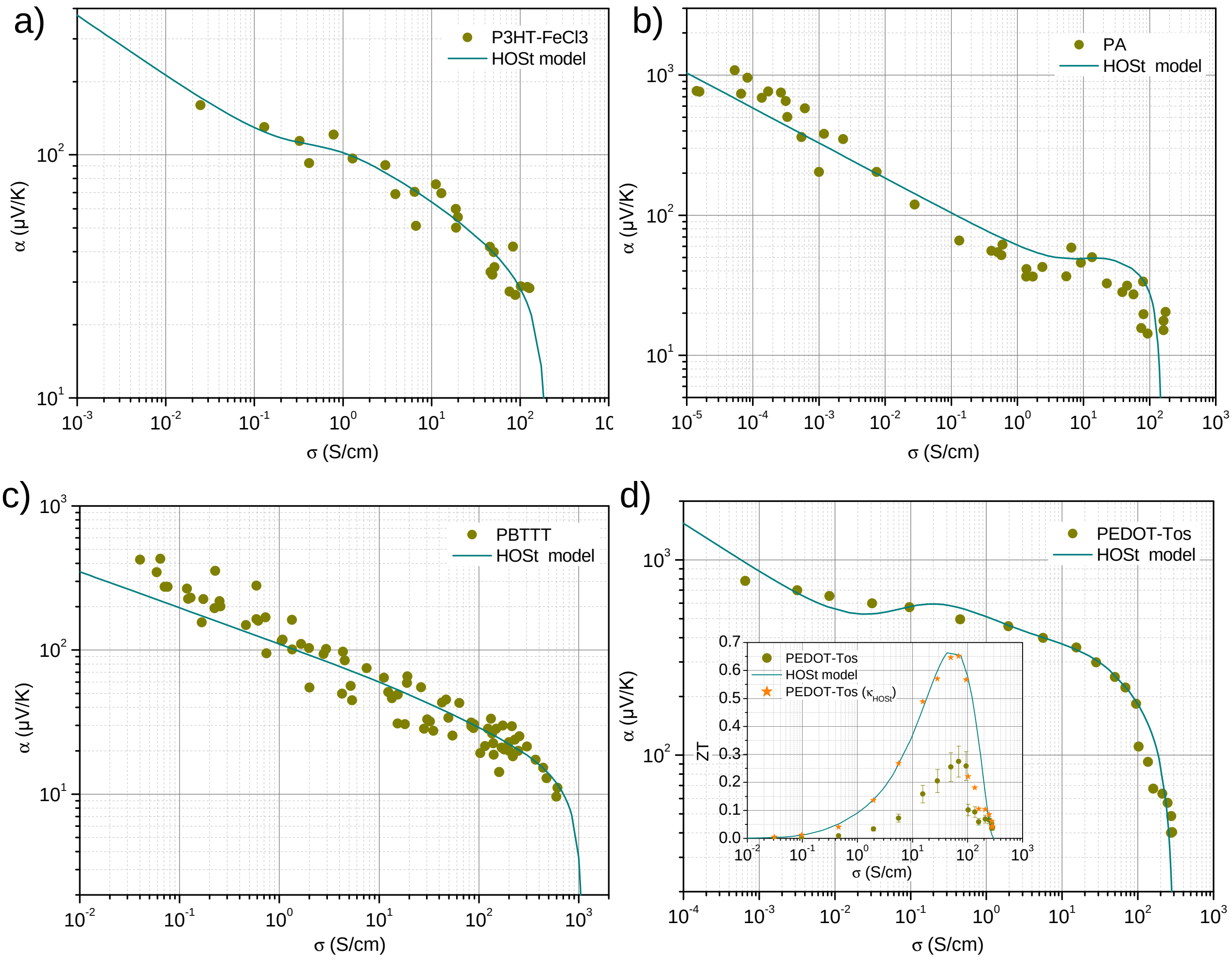

Figure S2: Thermopower as a function of the electrical conductivity in (**a**) P3HT-$FeCl_3$ [1], (**b**) PA [2-7], (**c**) PBTTT [8, 9] and (**d**) PEDOT-Tos [10]. The inset of the latter displays a comparison between the reported figure of merit ZT [10] and the calculated one according the HOSt model with the related transport parameters in the Table 1. Note that the reported ZT [10] has been inferred by using a constant thermal conductivity measured for the highest conducting film (0.37 W/m/K) which likely underestimates ZT since as shown according to the HOSt model, the thermal conductivity is expected to decrease with a decreasing σ. This explains why the ZT predicted here

according to the HOSt model is higher than the one inferred in [10]. As a matter of fact, the figure of merit which is here re-calculated by using the thermal conductivity expected in the frame of the HOSt model (PEDOT-Tos (κ_{HOSt})) reveals a suitable agreement.

As previously discussed the observation of the experimental curves α–σ in Fig. S2a-d allows to roughly classify the behaviors. Basically, the P3HT-$FeCl_3$ and the PA seem to belong to the case where $\sigma_{dis,\parallel}^{max} < \sigma_{c-o,\parallel} < \sigma_{c-o,\perp} < \sigma_{dis,\perp}^{max}$, the PBTTT to $\sigma_{dis,\parallel}^{max} < \sigma_{c-o,\parallel} < \sigma_{dis,\perp}^{max} < \sigma_{c-o,\perp}$ and the PEDOT to $\sigma_{c-o,\parallel} < \sigma_{dis,\parallel}^{max} < \sigma_{c-o,\perp} < \sigma_{dis,\perp}^{max}$. The reported transport parameters in the Table 1 confirm these conclusions for the P3HT-$FeCl_3$, the PA and the PBTTT while a more complex situation is found in the PEDOT with $\sigma_{c-o,\parallel} < \sigma_{dis,\parallel}^{max} < \sigma_{c-o,\perp} < \sigma_{dis,\perp}^{max}$. In the latter case, the effects of both the parallel and perpendicular components strongly couple by making the thermopower variation very weak at low conductivity. In all compounds it is found $\sigma_{dis,\perp}^{max} > \sigma_{dis,\parallel}^{max}$ which could suggests that the expected shape anisotropy is met with a width of the disordered domains much smaller than their extension. On the other hand, the transport parameters found in the PBTTT are qualitatively consistent with those determined in our investigations (see the main text) even if some of them differ quantitatively, likely because of the very different doping method based on transistor with TFSI− gate [1,8,9].

CP	Ordered domains			Disordered domains					γ	χ
	σ_0 (S/cm)	$\kappa_{ord,lat}$ (W/m/K)	β	$\sigma_{dis,\perp}^{max}$ (S/cm)	$\sigma_{c-o,\perp}$ (S/cm)	$\sigma_{dis,\parallel}^{max}$ (S/cm)	$\sigma_{c-o,\parallel}$ (S/cm)	$\kappa_{dis,lat}$ (W/m/K)		
PBTTT*	0.013	0.2	4	1300	4000	57	13	0.033	0.5	0.55
**P3HT-$FeCl_3$**	0.013	0.2	4	47.9	126	1.2	0.63	0.033	0.5	0.55
**PA**	0.013	0.2	4	34.7	41.7	30.2	7.2	0.033	0.5	0.55
**PBTTT**	0.013	0.2	4	174	437	0.066	275	0.033	0.5	0.55
**PEDOT-Tos**	2.4	0.2	4	69.2	7.24	0.251	0.0048	0.033	0.5	0.55

Table 1: Summary of the transport parameters inferred from the HOSt model as used in Fig. S1 for the non-oriented CP as P3HT-$FeCl_3$ [1], PA [2-7], PBTTT [8, 9] and PEDOT-Tos [10]. The *PBTTT** is the non-oriented CP investigated in the main text. The alignment degree and the crystallinity ratio have been kept constant.

Finally, let's briefly discussed the transport parameters of the PEDOT-Tos reported in the Table 1. As aforementioned they lie in a complex regime with $\sigma_{c-o,\parallel} < \sigma_{dis,\parallel}^{max} < \sigma_{c-o,\perp} < \sigma_{dis,\perp}^{max}$ which implies a coupling of both parallel and perpendicular components in the α–σ curve in Fig. S2d. This explains the observed weaker variation of the thermopower in the low conductivity regime compared with those seen in other CP in Fig. S2a-c. Furthermore, It has been necessary to increase the transport parameter of the ordered domains σ_0 up to nearly 2.4 S/cm in contrast to the other CP for which it remains the same. This appears to be the reason for the higher thermoelectric effciciency in PEDOT-Tos compared to the other CP. If one considers the previously reported microscopic model which explains the unconventional scaling power law measured in CP [11], the σ_0 parameter is related to microscopic quantities involving the quasiparticles velocity, the density of states and the scattering time as defined below according to their general power laws.

$$\tau_E = \tau_0 \left(\frac{E}{E_\tau} \right)^\theta \qquad v_{x,E}^2 = v_0^2 \left(\frac{E}{E_v} \right)^\nu \qquad g_E = g_0 \left(\frac{E}{E_g} \right)^{\gamma_g} \quad \Rightarrow \quad \sigma_0 = q^2 g_0 v_0^2 \tau_0 \frac{(k_B T)^s}{E_\tau^\theta E_v^\nu E_g^{\gamma_g}}$$

with k_B the Boltzmann constant, q the elementary charge and the scaling exponent $s = \theta + \nu + \gamma_g$. As previously demonstrated, if one considers pseudo-relativistic massless quasiparticles with

$E_k = \hbar v_F k$ (the exponent $\nu = 0$), the three dimensional density of states $g_E = \frac{E^2}{\pi^2 (\hbar v_F)^3}$ ($\gamma_g = 2$) and the scattering by unscreened ionized impurities ($\theta = 2$ and $\tau_0 \propto 1/v_F$), it follows that s=4 and the transport parameter σ_0 can be written as below.

$$\sigma_0 = \frac{q^2 \tau_0}{\pi^2 \hbar^3 v_F} \frac{(k_B T)^4}{E_\tau^2} \propto \frac{\tau_0}{v_F} \propto \frac{1}{v_F^2}$$

Since E_τ mainly depends on the impurities density which might be ascribed to the dopants, it should not be so different from one CP to another. Thus, the remaining dependence in σ_0 involves the square of the Fermi velocity v_F as shown above. This implies that a low v_F can increase significantly σ_0. By considering the values reported in the Table 1, it would mean that the Fermi velocity in the PEDOT-Tos could be about 13 times lower than in the other CP. Whereas this could provide a piece of explanation for the exceptional thermoelectric efficiency of the PEDOT compared to the other CP, one must emphasize that it could also result from another specific characteristics such as the anisotropy or a combination of both.

References.

1. Gregory, S. A., Hanus, R., Atassi, A. J., Rinehart, M., Wooding, J. P., Menon, A. K., Losego, M. D., Snyder, G. J. & Yee, S. K. Quantifying charge carrier localization in chemically doped semiconducting polymers. *Nature Materials*, **20**, 1414 (2021). https://doi.org/ 10.1038/s41563-021-01008-0

2. Przybylski, M., Bulka, B. R., Kulszewicz, I. & Proń, A. Electrical transport properties of polyacetylene tetrachloroferrate. *Solid State Commun.* **48**, 893–896 (1983). https://doi.org/10.1016/0038-1098(83)90143-6

3. Kaiser, A. B. Thermoelectric power and conductivity of heterogeneous conducting polymers. *Phys. Rev. B* **40**, 2806–2813 (1989). https://doi.org/10.1103/physrevb.40.2806

4. Jones, T. E., Ogden, T. R., McGinnis, W. C., Butler, W. F. & Gottfredson, D. M. Electronic properties of polyacetylene doped with FeCl3. *J. Chem. Phys.* **83**, 2532–2537 (1985). https://doi.org/10.1063/1.449299

5. Chiang, C. K. et al. Electrical conductivity in doped polyacetylene. *Phys. Rev. Lett.* **39**, 1098–1101 (1977). https://doi.org/10.1103/PhysRevLett.39.1098

6. Chiang, C. K. et al. Conducting polymers: halogen doped polyacetylene. *J.Chem. Phys.* **69**, 5098–5104 (1978). https://doi.org/10.1063/1.436503

7. Park, Y.-W., Heeger, A. J., Druy, M. A. & MacDiarmid, A. G. Electrical transport in doped polyacetylene. *J. Chem. Phys.* **73**, 946–957 (1980). https://doi.org/10.1063/1.440214

8. Tanaka, H. et al. Thermoelectric properties of a semicrystalline polymer doped beyond the insulator-to-metal transition by electrolyte gating. *Sci. Adv.* **6**, eaay8065 (2020). https://doi.org/10.1126/sciadv.aay8065

9. Thomas, E. M., Popere, B. C., Fang, H., Chabinyc, M. L. & Segalman, R. A. Role of disorder induced by doping on the thermoelectric properties of semiconducting polymers. *Chem. Mater.* **30**, 2965–2972 (2018). https://doi.org/10.1021/acs.chemmater.8b00394

10. Bubnova, O., Khan, Z. U., Malti, A., Braun, S., Fahlman, M., Berggren, M. & Crispin, X. Optimization of the thermoelectric figure of merit in the conducting polymer poly(3,4-ethylenedioxythiophene). *Nature Materials*, **10**, 429 (2011). https://doi.org/10.1038/nmat3012

11. Lepinoy, M., Limelette, P., Schmaltz, B. & Tran Van, F. Thermopower scaling in conducting polymers. *Sci Rep*, **10**, 8086 (2020). https://doi.org/10.1038/s41598-020-64951-z
